# The end of urban sprawl? Internal migration across the rural‐urban continuum in Switzerland, 1966−2018

**DOI:** 10.1002/psp.2621

**Published:** 2022-10-18

**Authors:** Mathias Lerch

**Affiliations:** ^1^ Ecole Polytechnique Fédérale de Lausanne (EPFL) Lausanne Switzerland

**Keywords:** Europe, internal migration, re‐urbanisation, sociodemographic differentials, urban sprawl, urban‐rural continuum

## Abstract

In high‐income countries, migration redistributed populations from congested city centres into the sparsely populated outskirts, raising challenges to environmental and population health and the conservation of biodiversity. We evaluate whether this periurbanisation process came to a halt in Switzerland by expecting a decline in internal migration and a renewed residential attractiveness of urban agglomeration centres (i.e., re‐urbanisation)—two recent trend changes observed in Europe. Relying on data from censuses, registers and surveys, we describe trends in the intensity, geography and sociodemographic differentials of migration across consistently defined urban agglomeration density zones between 1966 and 2018. Although the overall intensity of migration declined, the rate increased among the working age population in part because of the societal diffusion of tertiary education. The dominant urban‐bound migration flows are increasingly confined within agglomerations over time. After the diffusion of periurbanisation down the city hierarchy between 1966 and 1990, we observe the emergence of re‐urbanisation in some agglomerations and sociodemographic groups around 2000. However, this phenomenon has been temporarily inflated by period‐specific transformations in Swiss society. More recently, the process of periurbanisation intensified again and expanded more and more beyond official agglomeration borders.

## 1 INTRODUCTION

Population movements within countries, hereafter referred to as internal migration, play a crucial role in the spatial distribution of demographic and economic potential in contemporary contexts of low fertility. The dominant pattern of internal migration in high‐income countries in the second half of the 20th Century redistributed population from congested city centres into the sparsely populated outskirts—a process referred to as urban sprawl, counter‐ or periurbanisation (Champion, 2001; Shaw et al., 2020). This spatial extension of urban agglomerations raises challenges to population and environmental health as well as to the conservation of biodiversity (OECD, 2018). Increased commuting between residence and work places produces higher greenhouse gaz emissions, as well as adverse health outcomes in individuals due to air and noise pollution and limited time for physical exercise. The expansion of low density built‐up environments also increases energy consumption, may occur at the expense of forests, decimates invertebrate species, lowers ground‐water reserves and affects population health through heat island effects.^1^ In line with the United Nations' Sustainable Development Goal 11 (‘make cities and human settlements inclusive, safe, resilient and sustainable’),^2^ local authorities have issued various directives to limit urban sprawl. We question whether the process of periurbanisation came to a halt in Switzerland by investigating trends and patterns of internal migration across agglomeration density zones over the last 50 years.

This study addresses two important issues arising from recent international research that challenged established models of internal migration and urbanisation (Bell et al., 2018; Salvati et al., 2019). We question whether the intensity of internal migration has declined, and whether its geographic pattern has switched from the centrifugal process of periurbanisation to a move back to central areas of agglomerations (i.e., re‐urbanisation), leading to a renewed spatial concentration of urbanisation. The present case study on Switzerland—a highly urbanised and developed country in Europe—contributes to the existing evidence in two ways. To identify the spatial diffusion of urban migration patterns, we adopt a geographically detailed and internationally comparative perspective of the rural‐urban continuum defined consistently over time and across space. The long‐term perspective of this analysis, provides the keys to understand the importance of recent changes in migration and urbanisation in the context of the longstanding trends that continue to unfold.

After reviewing the recent evidence on internal migration and population redistribution, we present the data and methods. This is followed by a countrywide description of trends, sociodemographic differentials and the spatial focus of internal movements in Switzerland. We also map changing geographic patterns of migration in the 79 urban agglomerations and identify the population groups driving those trends. The paper concludes by a summary of the results and a discussion of their relevance for understanding the process of urbanisation in high income countries.

## 2 BACKGROUND & RECENT EVIDENCE

The hypotheses of a mobility transition (Cooke et al., 2018; Skeldon, 2019; Zelinsky, 1971) and differential urbanisation (Champion, 2001; Geyer & Kontuly, 1993; Kontuly & Geyer, 2003) propose a patterned spatiotemporal diffusion and changing motives of migration during the transformation from a predominantly rural to an essentially urban and developed society. In early stages of this urbanisation process, the rising economic centre—the primate city—pulls migrants looking for new income opportunities. With the regional diffusion of development, subnational income disparities attenuate, redirecting migrants towards lower‐ranked cities. As a consequence, the city hierarchy diversifies. When the society is predominantly urban, rural‐to‐urban migration diminishes. Intercity and within‐city flows are then expected to dominate migratory movements. We investigate whether the migration patterns of Switzerland correspond to this late stage of population redistribution. The proportion of inhabitants living in urban areas indeed increased from 50% to more than 80% between 1960 and the early 2010s (BFS, 2017; Cunha & Both, 2004).

Massive migration into cities led to urban and industrial congestion effects in central areas. Consequently, jobs have been delocalised outward. People followed this move, thereby spatially extending the city borders [a process referred to as suburbanisation; (Champion, 2001)]. With the shift from an industrial to a postindustrial economy starting in the 1960s, a second phase of urban sprawl emerged due to multiple factors (Champion, 1989; OECD, 2018; Rubiera‐Morollón & Garrido‐Yserte, 2020; Shaw et al., 2020). Rising income per capita increased aspirations for more housing space (which is scarce and/or expensive in central areas of agglomerations), as well as for environmental amenities in less congested and more natural settings. The improvement of transport and communication technologies reduced the significance of distance to the workplace as a residential determinant. The diffusion of cars and large‐scale investments in road and public transport infrastructure enabled people to commute over longer distances.

Thus, the second phase of urban sprawl extends into formerly rural areas of low population density, located on the more distant urban periphery; a process referred to as peri‐ or counter‐urbanisation (Champion, 1989).^3^ This changing geography of migration over the stages of urbanisation—from centripetal flows into central areas of urban agglomerations to centrifugal movements towards the outskirts—has been confirmed by recent cross‐sectional data in various world regions (Charles‐Edwards et al., 2017; Rees et al., 2017; Rodriguez, 2007). Between the 1960s and the 1990s, Switzerland experienced intense periurbanisation of highly skilled individuals and upper‐class families, leading to population decline in the centres of agglomerations (Cunha & Both, 2004). The present study traces the onset and spatial diffusion of this process over the last 50 years.

However, recent international research suggests a potential slowing down (and ultimately the end) of periurbanisation in highly urbanised settings. Firstly, the intensity of internal migration has surprisingly declined in many countries across the world since 1980—although Europe was less affected by this phenomenon (Alvarez et al., 2021; Bell et al., 2018; Cooke, 2013). The drivers of this recent observation are not yet established. Kalemba et al. (2021) showed how population ageing determined the decline in migration within Australia over time. This trend has been only partly counterbalanced by an increasing share of geographically more mobile groups in the population, such as international immigrants and highly skilled adults in working ages. Education indeed raises aspirations for mobility, increases its potential benefits and helps overcoming its financial barriers (DeHaas, 2010; Sjaastad, 1962). In Switzerland as elsewhere, individuals with more skills, better pay and higher professional status migrate more than the lower social strata (Bernard & Bell, 2018; Carnazzi‐Weber & Golay, 2005; Charton & Wanner, 2001; Zufferey, 2020). Although Switzerland ranks among the countries with the highest intensities of internal migration in the world (Bell et al., 2015), we lack evidence on trends according to people's family stage and educational attainment at fine spatial scales.

A second unexpected finding in the literature relates to the renewed population growth in central areas of urban agglomerations since the turn of the 21th century in a number of European countries (such as in Italy, Germany, the Netherlands, Spain, the United Kingdom, etc.) (Dembski et al., 2021; Halbac‐Cotoara‐Zamfir et al., 2020; Kabisch & Haase, 2011; Salvati et al., 2019).^4^ The drivers of this so‐called re‐urbanisation process are multiple, including market forces, the individuals' changing residential preferences and urban planning (Dembski et al., 2021; Rérat, 2012; Salvati et al., 2019). With economic globalisation, the rising economic sector of specialised services with high added value requires centrality to benefit from frequent interactions among agents and enhanced access to international transport and communication infrastructure. To ensure international competitiveness, many cities have also regenerated central areas of agglomerations by constructing new business, cultural and residential districts. This has led to a gentrification process, which substitutes higher‐class inhabitants to socially disadvantaged residents. Furthermore, an increasing share of the youth is attracted by central places in the context of the boom in higher‐level education. Over the last decade, people also increasingly postponed the onset of family formation to later stages of their life course. As a consequence, adults can enjoy downtown city‐life over a longer period of time before moving into the urban periphery to raise children. Finally, governments concerned with the environmental impacts of urban sprawl increasingly implement urban containment measures that aim at constraining the inhabitants' locational choices within existing built‐up areas.

Following three decades of population decline, central areas of Swiss agglomerations experienced renewed population growth since the turn of the new Century. This was driven primarily by the arrival of an increasing number of international immigrants and, secondly, by the youth and the young and highly skilled working‐age adults (Rérat, 2012, 2019). Switzerland's revised Spatial Planning Act of 2014 promotes this change in settlement patterns to densify cities and mitigate the environmental risks of urban sprawl. Many local and cantonal authorities have defined a moratorium on the zoning of new constructible land areas and issued directives to constrain future construction activities within the agglomeration extents (rather than beyond).

Assessments of urbanisation patterns from an land‐use (rather than a demographic) perspective, by contrast, highlight a continuous expansion of settlement areas in Switzerland, although the pace slowed down recently (BFS, 2019). The proportion of the urban residents that lives in zones with less than 1.5 thousand inhabitants per square kilometre continues to decrease at an intensifying pace (OECD, 2018). Population re‐urbanisation in Switzerland actually emerged alongside continuous demographic growth in the urban periphery, which remained the dominant form of urbanisation until recently (Rérat, 2012, 2019).

The question is thus whether re‐urbanisation is about to supersede periurbanisation. Previous research in Switzerland has emphasised international migration flows as the main driver of renewed population growth in central areas of agglomerations (Rérat, 2012, 2019). Agglomeration centres obviously constitute gateways for new immigrants (Cunha & Both, 2004), especially since the late 1990s when highly skilled individuals working in the knowledge‐based sectors of the economy have dominated the inflows (Wanner & Steiner, 2018). However, this demographic contribution becomes increasingly temporary for two reasons. First, more than half of recent immigrants spent less than 6 years in Switzerland to develop their professional career before moving on to another country or returning back home (Fioretta & Wanner, 2017; Wanner, 2020). Second, those migrants, who integrated over a longer period of time in Swiss society, are characterised by higher levels of internal mobility than the natives (Charton & Wanner, 2001) and have adopted similar residential preferences for periurban zones (Lerch & Wanner, 2010). We argue that sustainable re‐urbanisation over the long term depends on the redistribution of the resident population through internal movements, rather than on international migration flows.

Yet we lack evidence for a renewed shift in the geography of internal migration—from a centrifugal pattern (leading to periurbanisation) back to a centripetal one (driving re‐urbanisation). It remains unclear whether re‐urbanisation is consolidating or whether the urban containment measures only had a limited impact. In this context, we evaluate whether a potential decline and a change in the geography of internal migration has led to the end of periurbanisation in Switzerland.

## 3 METHODOLOGY

We describe trends, sociodemographic differentials and spatial patterns of migration across agglomerations and the rural‐urban continuum to understand better the dynamics of population redistribution in Switzerland between 1966 and 2018. Methodological details are discussed below.

### 3.1 Data and definitions

We rely on individual‐level data from the full censuses 1970, 1980, 1990 and 2000, and the population register 2015−2018, which all provide information on migration status, and the municipalities of origin and destination. The censuses also include information on educational attainment. As this information is not available in the population register, we further mobilise the pooled annual waves 2015−2018 of the structural survey (which is administered to a 3%‐sample of the registered population). The censuses provide the individuals' economic or de facto place of residence (i.e., weekly residents are enumerated at their place of work or study, rather than where they are registered). The register and the survey, by contrast, provide information on the legal residence. The discrepancy between the two approaches may be particularly important for young people. The concept of usual residence is ambiguous at that age, especially for students spending the workweek in the place of higher‐level education, while remaining registered in their parents' household. We are unable to adjust the census data to the legal definition in the register/survey data (because legal place of the migrants' origin is unknown). The uncertainty of the quality of information on usual place of residence calls for caution in the interpretation of the results—especially for the youth.^5^

Based on the comparison of an individual's place of residence at the time of data collection with the self‐declared place of residence 5 years earlier (i.e., migrant transitions), we analyse internal migration matrices for the 5‐year periods 1966−1970, 1976−1980, 1986−1990, 1996−2000. For the last decade, we estimated matrices referring to four overlapping 5‐year periods (i.e., 2011−2015, 2012−2016, 2013−2017 and 2014−2018) and averaged the results for an average 5‐year period between 2010 and 2018, to limit uncertainty related to the sampling of respondents to the structural survey.

In this analysis, we do not account for international migration flows for the reasons stated earlier and because our data only allow to observe international inflows, not the outflows. We focus on internal migration of the resident population as enumerated or registered at the dates of the respective data collections. Recent international migrants who arrived in the immediately preceding 5 years are excluded, but longer‐term immigrants are included in the resident population.

During the observation period, Switzerland experienced sustained population growth from 5.8 million inhabitants in the mid‐1960s to 8.6 million in 2019, as well as strong economic growth [the gross domestic product per capita (GDPc) rose from 13,000 to 84,000 CHF at current prices]. However, the trend in economic development was discontinuous, with periods of sustained growth and intermittent phases of stagnation and crisis. Our 5‐year periods of observations can nevertheless be compared with each other, as they were are all marked by a positive economic context. Even in the last period observed (the aftermath of the global financial crisis), GDPc continued to grow—although at a significantly reduced pace.

### 3.2 Spatial classification

**Figure 1:**
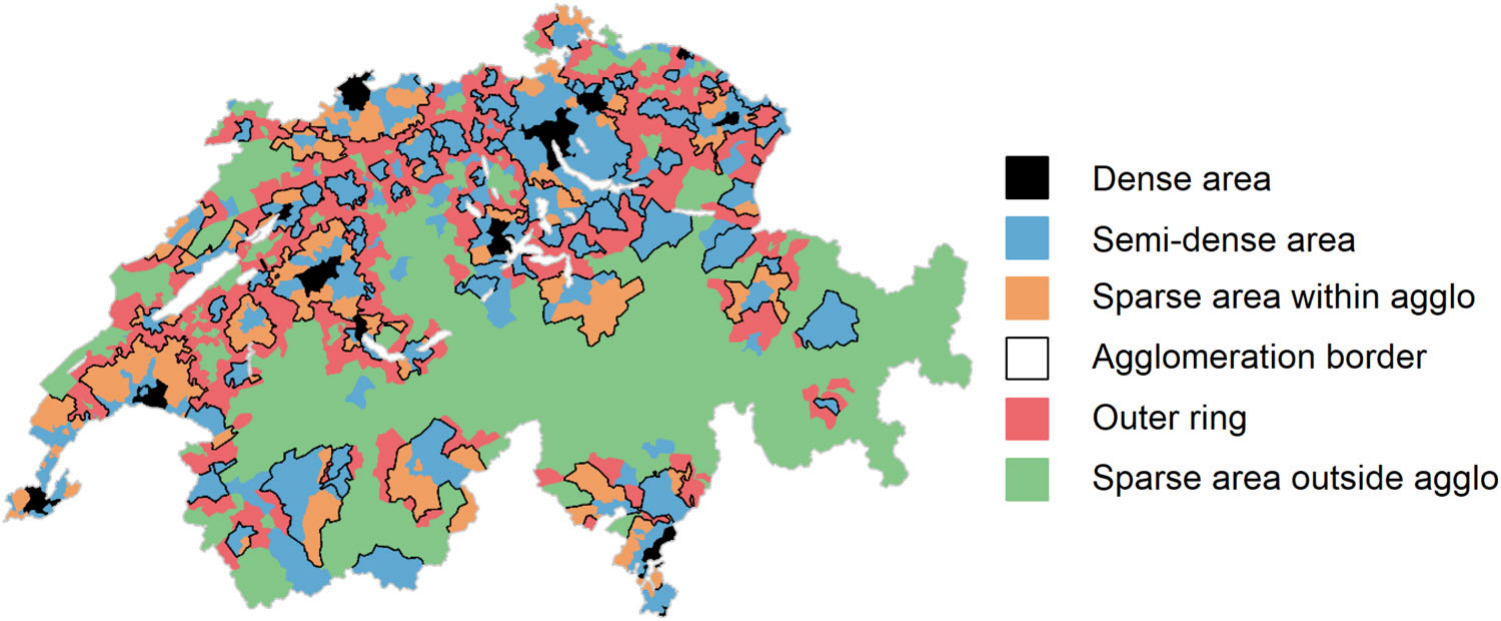
Constant definition of agglomeration density areas and ring areas (as of 2018), Switzerland. *Sources*: BFS (2017) definition of the agglomeration extents and the urban density classes; author's spatial identification of outer agglomeration ring areas constituted of rural municipalities. ‘agglo’ = agglomeration; white coloured polygons with borders in light‐grey represent lakes.

To analyse migration across the rural‐urban continuum, we regroup municipalities into urban agglomerations (i.e., functional urban areas, comprising several contiguous local administrative units with integrated labour markets). The Swiss Federal Statistical Office (SFSO) defined the spatial extent of these agglomerations based on geolocalised data on the continuity and form of the built‐up area, population density, as well as on the scale of over‐night stays and commuting to the agglomeration's central municipality [which is represented by an official city—i.e., a municipality with at least 10,000 inhabitants; (BFS, 2017)]. The 79 distinct urban agglomerations are shown in Figure 1.

We further disaggregate urban zones in accounting for the rural‐urban continuum of built‐up areas. We rely on the SFSO's operationalization of Eurostat's new ‘degree of urban’ classification based on 300 x 300 m grid cells of the Swiss territory, aggregated at the municipality level (BFS, 2017). This internationally comparative definition of the rural‐urban continuum has been adopted by the international community to promote comparative statistics on urbanisation worldwide. We disaggregate agglomeration zones in three population‐density classes, that are organised in the form of concentric rings around the central municipality: densely populated areas or core cities (i.e., central municipalities with more than 500 inhabitants per km^2^), semidensely populated places (corresponding to suburbs with 200−500 inhabitants per km^2^), as well as the sparsely populated areas within agglomerations (corresponding to periurban zones) and those located outside of the agglomerations (see Figure 1).

If periurbanisation will not come to a halt, agglomerations will further extend into rural areas. We therefore defined an additional agglomeration zone, as constituted by a concentric ring of officially designated rural municipalities that directly border the agglomerations. Accounting for these extended agglomeration borders provides an interesting outlook on the potential future urban territory of Switzerland. In the case of continuous periurbanisation, the Swiss plateau on the North of the Alps will constitute one single and continuously urbanised mega‐urban area, rather than a set of spatially independent agglomerations as observed in 2018. This scenario is at odds with the largely accepted urbanisation scenario of ‘metropolisation’. The latter territorial vision aims to ensure the Swiss cities' international competitiveness in a globalised economy, while limiting the negative environmental consequences of sprawl. This is ensured by a set of distinct metropolitan regions—each of them including a large polycephal urban area centred around the most dynamic city and containing its inhabitants within a densely populated built environment (Cunha & Both, 2004; Kaufmann, 2012).

The analysis below refers to the extended urban agglomeration extents, thereby adding an additional differentiation of the urban‐rural continuum of space. Hence internal migration is defined as a change in place of residence across 199 spatial zones, including the concentric agglomeration density zones (dense, semidense and sparse areas) and the ring areas of the 79 distinct agglomerations, as well as the remaining rural territory of Switzerland.

This spatial disaggregation of the Swiss territory as of 2018 is applied to the data for earlier years. In other words, we control for the rural‐urban reclassification of municipalities over the process of urban sprawl since 1966 to focus on migratory dynamics across consistently defined areas over time. Consistency in the spatial disaggregation was ensured by harmonising the municipality structure of the Swiss territory over the five decades under study in collaboration with the Statistical Office of the canton of Zurich, taking into account the administrative regrouping and/or splitting of municipalities, as well as the exchanges of territory between them—as reported by SFSO in its historical registry of municipalities (BFS, 2021).

In the analysis of sociodemographic differentials in migration, we distinguish the following population age groups: children aged less than 15, the youth and young adults aged between 15 and 24, the working age population aged 25−64 (with an additional distinction of the young and mature subgroups aged, respectively, 25−34 and 35−64) and the elderly aged 65 or more. This enables us to investigate how migration in different stages of the life course evolved over time. As a proxy for social status, we rely on the level of completed education, distinguishing (i) the primary level including individuals with at best a compulsory school diploma (9 years of schooling), (ii) the secondary level containing persons with a professional training or a high school diploma and (iii) the tertiary level including those with a university degree. This information is comparable over the successive census and survey rounds only for the population aged between 25 and 64 years. The analysis relies on descriptive statistics, cartographic methods and decomposition analysis.

## 4 RESULTS

### 4.1 Trends in internal migration

**Figure 2:**
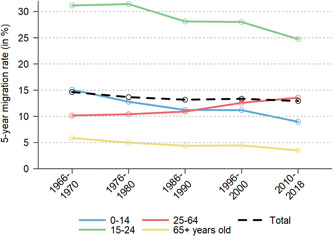
Crude rate of migration (over 5‐year periods) across the city hierarchy and the rural‐urban continuum, by age group, Switzerland 1966−2018. *Sources*: Population Censuses 1970, 1980, 1990, 2000, Population Register and Structural Survey 2015−2018.

To investigate whether the intensity of internal migration has declined in Switzerland, Figure 2 shows the trend in the crude internal migration rate across the urban hierarchy and the agglomeration‐specific density zones. The 5‐year rate of migration of the total population indeed continuously declined between 1966 and 2018, but only slowly from 14.63% to 12.91%.

The population aged 15−24 has the highest level of internal migration, but also experienced the strongest decline from 31.15% to 24.7% over time. Family migration is also decreasing, as evinced by the falling rate among children under 15 years of age, who usually move alongside their parents. Retirees move less over time, too (from 5.97% to 3.51%).

**Figure 3:**
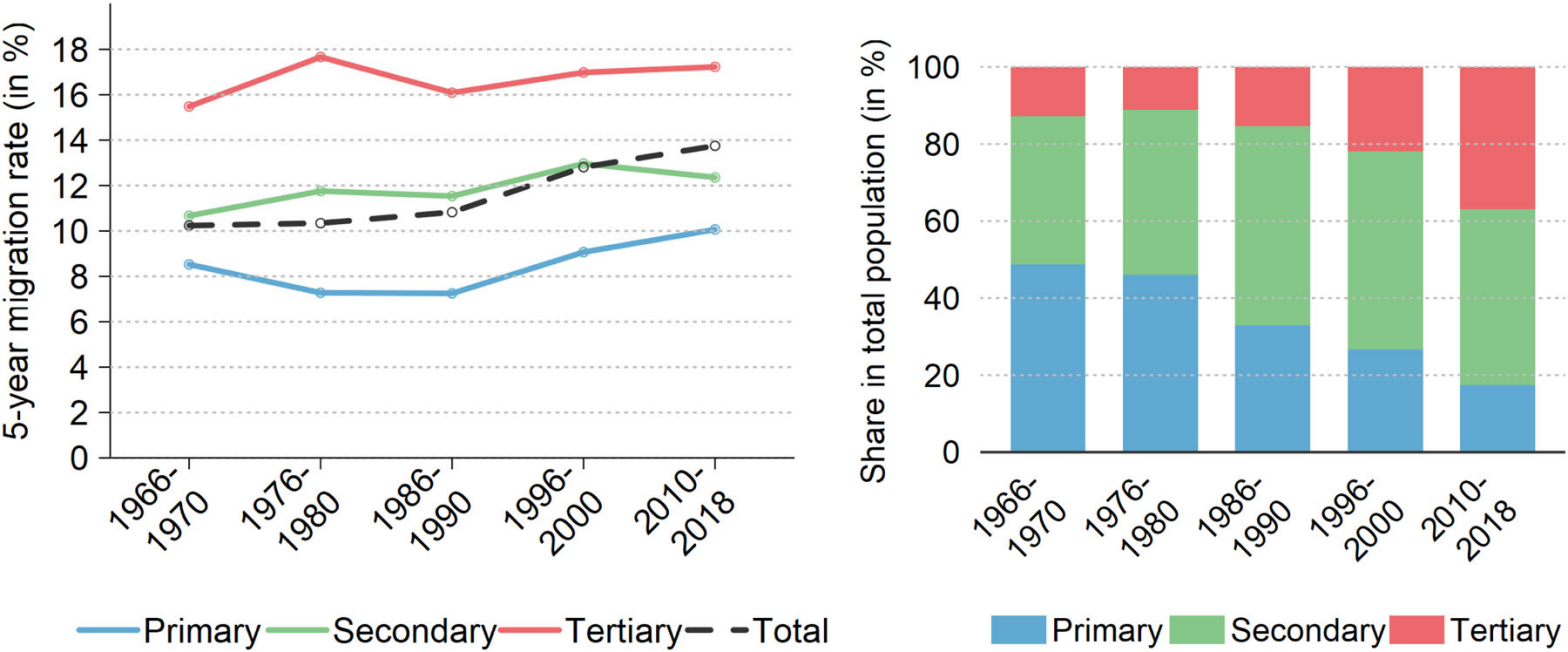
Education‐specific crude rates of migration across the city hierarchy and the rural‐urban continuum (left‐hand panel), as well as population distribution by education (right‐hand panel), population aged 25−64 years, Switzerland 1966−2018. *Sources*: Population Censuses 1970, 1980, 1990, 2000, Population Register and Structural Survey 2015−2018.

However, internal migration increased in the working age population (25−64 years), especially from the mid‐1980s on. Behavioural changes and the structural recomposition of this population explain the trend. As shown in Figure 3, the migration rates increased in all educational groups. The tertiary educated population moves up to twice as frequently when compared to lower skilled groups. In addition, the highly skilled are increasingly represented in the working age population: from only 12.72% in 1966−1970 to 36.83% in 2010−2018, when the lowest skilled group represented only 17.45% (down from 48.72%). Diffusion of higher‐level education in Swiss society played a crucial role in pushing up internal migration in the working age population. This attenuated the declining trend in the total population.

### 4.2 The Swiss‐wide urban geography of migration

Switzerland's advanced stage of urbanisation conjectures major inter‐ and intra‐agglomeration movements versus a marginal rural exodus. Urban‐bound migrants indeed clearly dominate, with a rather stable 82%−84% of migrants over time (not shown). Migrants moving between distinct agglomerations are the largest group, but their share is decreasing (from 58.11% in 1966−1970 to 47.69% in the last decade). Migrants increasingly move within agglomerations, especially between the late 1960s and 1970s (when the share increased from 23.54% to 29.75%, before reaching 33.97% in the last decade). While the minority group of rural‐to‐urban migrants declined over time (from 11.37% to 9.21%), the percentage of migrants moving in the opposite direction increased (6.97%−9.11%). This points to a spatial extension of periurbanisation in Switzerland.

**Figure 4:**
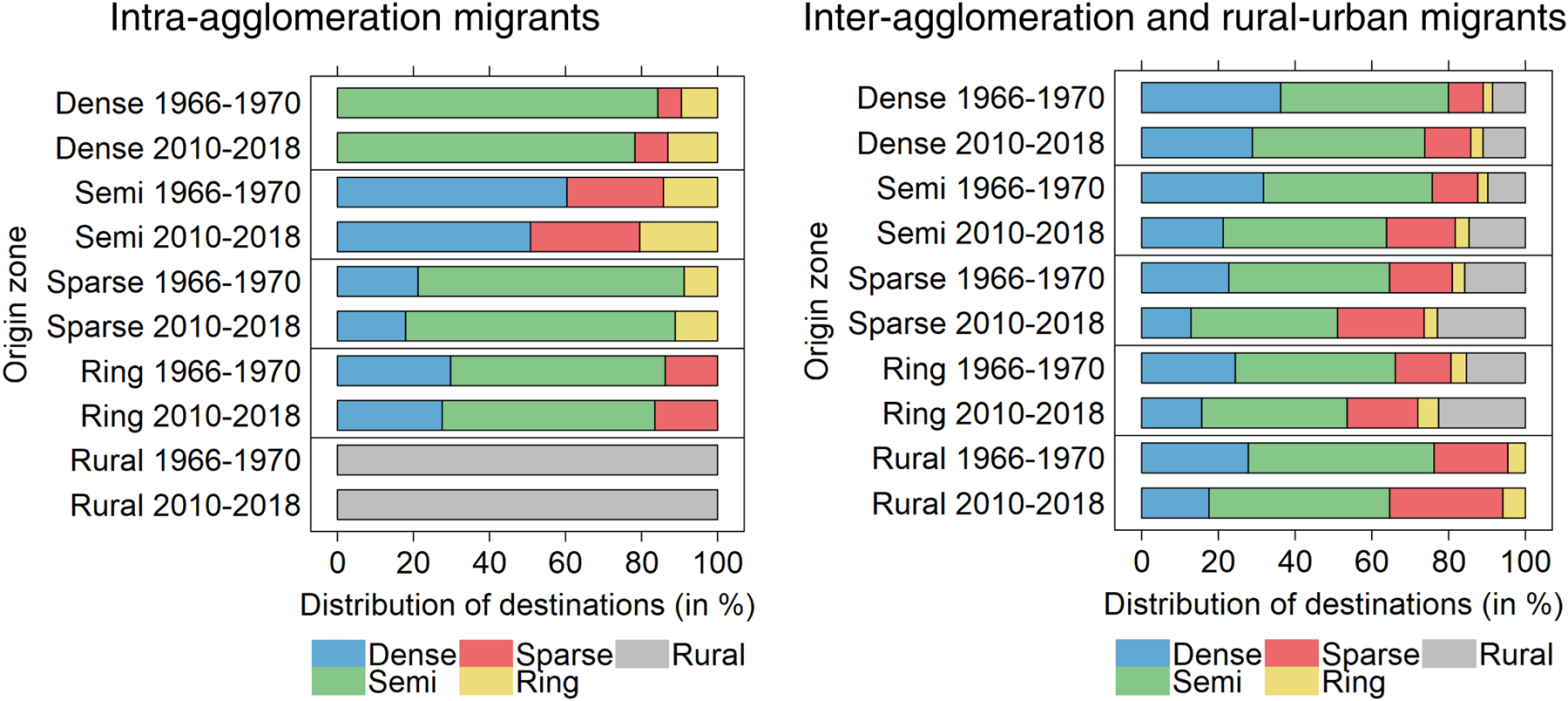
Relative distribution of the inter‐ and intra‐agglomeration migrants' destination zones, by origin zone, 1966−1970 and 2010−2018 (average 5‐year period), Switzerland. *Sources*: Population Censuses 1970, 1980, 1990, 2000, Population Register 2015−2018.

To evaluate whether the geography of migration shifted from a centrifugal pattern back to a centripetal pattern, Figure 4 shows the relative distribution of the (5‐year) migration flows' destination zones in 1966−1970 and 2010−2018 by origin zone for entire Switzerland. The left‐hand panel focuses on movements within agglomerations. Alongside their increasing importance, their spatial pattern became more (rather than less) centrifugal over time. Among the out‐migrants from dense areas, the percentage moving towards sparse and ring areas rose from 15.73% to 21.73%. The share of out‐migrants from semidense areas settling in more sparsely populated agglomeration zones increased from 39.62% to 49.21%. Within‐agglomeration migrants from sparse areas also increasingly focus towards the agglomeration ring (i.e., from 8.78% to 11.15% over the observation period).

The right‐hand panel of Figure 4 focuses on migrants between agglomerations. A centrifugal pattern emerged over time, too. Among the out‐migrants from dense urban zones, the share moving either to sparse and ring areas or into the rural territory increased from 20.05% to 26.23%. Among the out‐migrants from semidense zones, the proportion heading towards sparse, ring or rural areas increased from 24.26% to 36.16%. Migrants from sparse areas also increasingly move to similarly sparse, ring or rural zones over time. Even the rural out‐migrants more and more settle in sparse or ring zones (with increasing shares from 23.77% to 35.34%), rather than in the dense areas at destination.

Figure 5Net migration rates (over 5‐year periods) in the agglomeration density zones and ring areas, as well as in the remaining rural territory of Switzerland, 1966−2018. The official agglomeration borders (excluding the agglomeration ring areas) are drawn in white colour; white coloured polygons represent lakes. *Sources*: Population Censuses 1970, 1980, 1990, 2000, Population Register 2015−2018.

**Figure:**
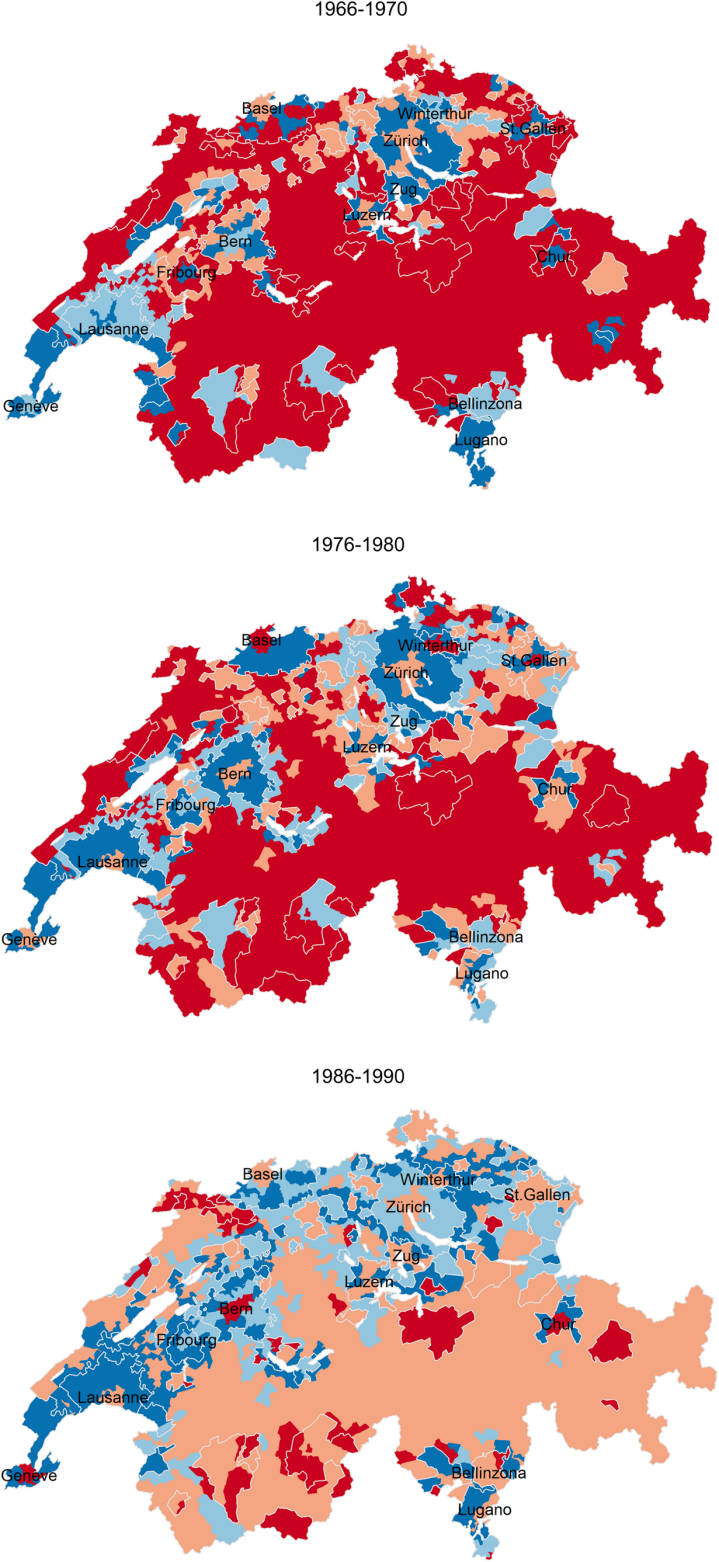

**Figure:**
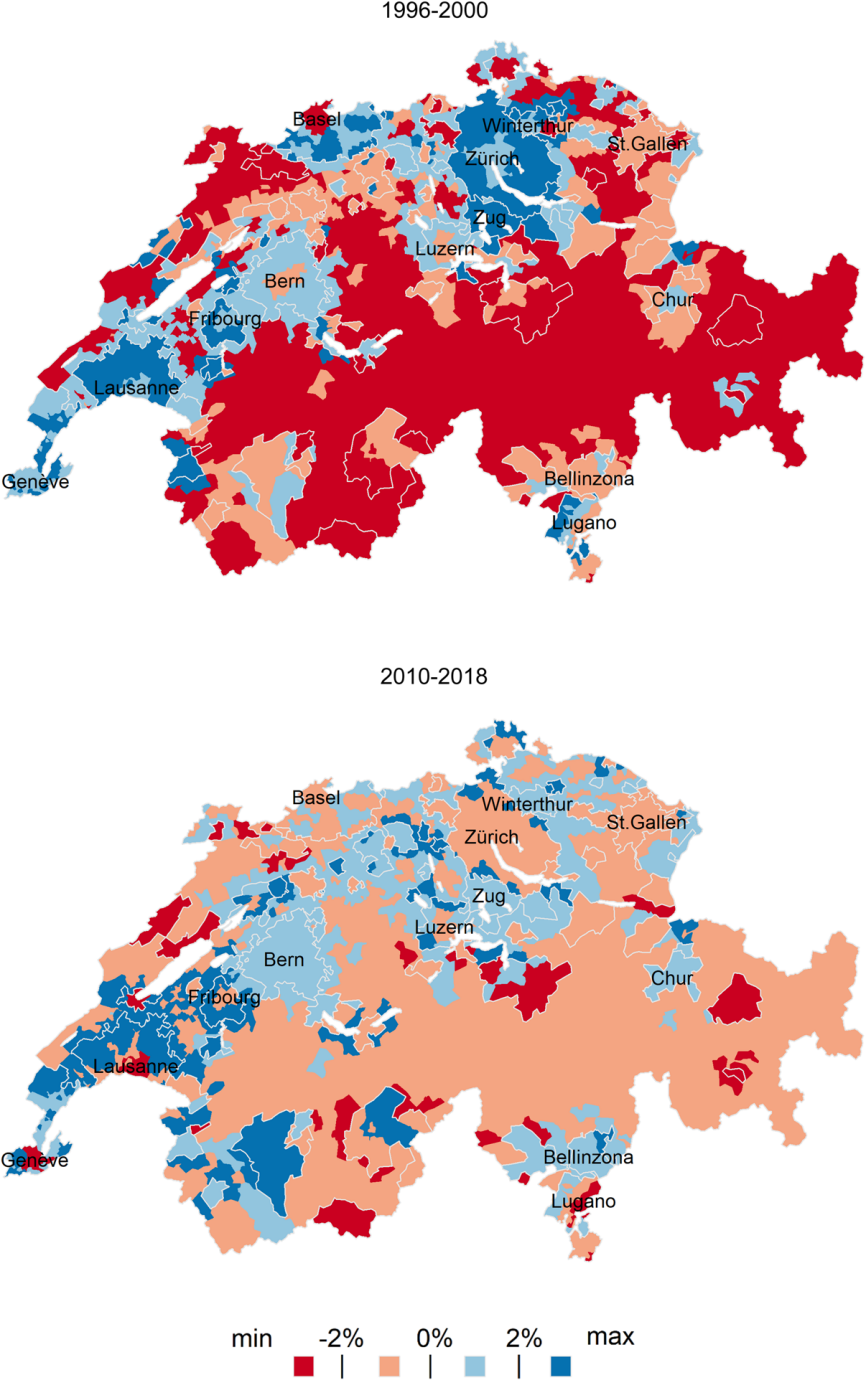

In sum, the dominant intra‐ and inter‐agglomeration migrants, as well as the marginal rural exodus, increasingly favour less densely populated zones in the periphery of agglomerations. This corresponds to a typical pattern of periurbanisation, rather than re‐urbanisation.

### 4.3 Detailed spatial patterns of net migration over time

To analyse the spatial diffusion of periurbanisation and the emergence and potential consolidation of re‐urbanisation across the 79 agglomerations, Figure 5 maps the 5‐year net migration rates of the agglomeration‐specific urban density zones and ring areas, as well as of the remaining rural territory of Switzerland for the five observation periods.

In the late 1960s, the rural exodus was still important. Migrants left the mountains, valleys and the countryside, including areas that constitute rural agglomeration rings in 2018. At the same time, periurbanisation emerged in Switzerland's two largest cities, Zurich and Basel: here, dense areas experienced a negative migration balance, which contrasts with the net migratory gains in the surrounding and less dense agglomeration zones. Urban sprawl diffused down the city hierarchy to almost all cities until 1990, while the migratory losses in rural areas slowed down. Rural fringes of agglomerations started to experience increasingly positive migration balances, which even surpassed the net migration levels of the semidense and sparse urban zones of Zurich as early as in 1986−1990. Yet we must consider that ring zones were still very sparsely populated in that period, which inflates in‐migration rates.

The spatial pattern of migration changed in 1996−2000. The dense areas of a number of agglomerations revealed a slightly positive net migration (e.g., in Zurich and several agglomerations in the West and South of Switzerland). This confirms the onset of re‐urbanisation. The new trend emerged alongside continued and sustained periurbanisation, which nevertheless expanded less in space when compared to the late 1980s. Migratory gains in the urban periphery were concentrated to a larger extent inside (rather than outside) of official agglomeration borders.

This re‐urbanisation phase was only short‐lived. In 2010−2018, the dense areas of almost all agglomerations reveal again negative migration balances, especially in the West and South of the country (Figure 5). In Zurich, Basel and Lausanne, net migratory losses now concern not only the dense zones, as in 1990, but also the semidense and sparse zones. In Zurich, net migration remained positive only in the extreme fringes of the agglomeration, many of which being still officially designated as rural (e.g., ring zones). Other large and intermediate‐sized agglomerations (such as Fribourg, Geneva, Lausanne, Luzern and Zug) also experience positive net migration beyond their current official borders.

### 4.4 Sociodemographic groups driving periurbanisation and re‐urbanisation

**Figure 6:**
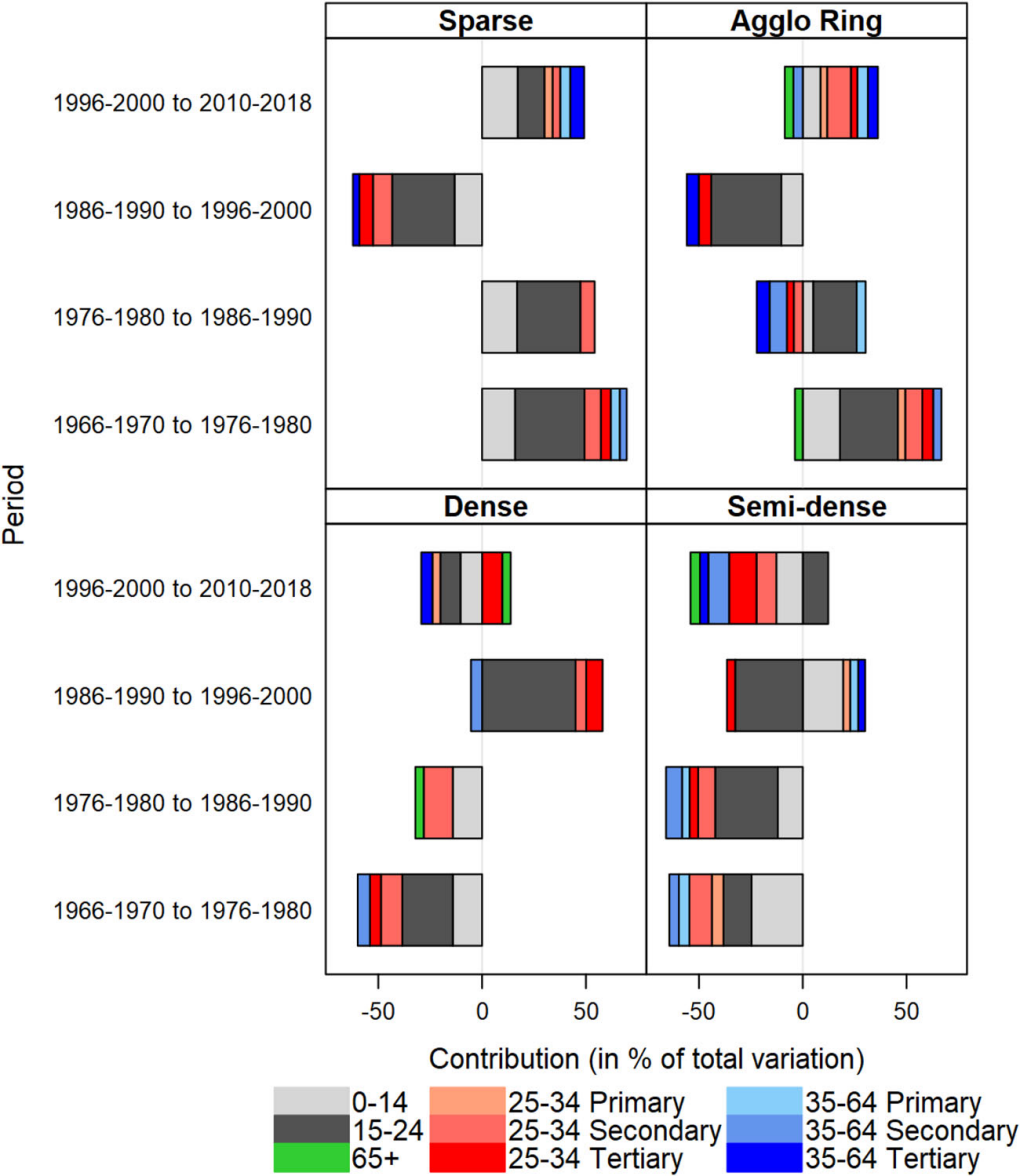
Contributions of behavioural changes in age‐ and education‐specific groups to the evolution in the crude 5‐year net migration rates by agglomeration density zone, Switzerland 1966−2018. *Sources*: Population Censuses 1970, 1980, 1990, 2000, Population Register and Structural Survey 2015−2018.

The geography of migration underwent successive shifts over time—from centrifugal flows out of dense areas into the sparser urban zones until 1990, to centripetal movements back to dense areas at the turn of the new Century, and resumed centrifugal flows beyond the official borders of agglomerations in the latest period. To evaluate the sources of temporal changes in the countrywide crude rates of net migration of different urban density zones, we decomposed the trends into the contribution of distinct sociodemographic groups (Figure 6).

We used Kitagawa's (1955) method of decomposition of rate changes. The 5‐year crude rate of net migration in a given agglomeration zone is a weighted average of the migration rates in different subgroups, with the weights being the groups' relative representations in the total population of that zone. In other words, changes in crude net migration in a given zone result as much from changes in group‐specific migration rates (e.g., behavioural effects) as from variations in the relative representation of more versus less migratory subgroups in the population (e.g., changes in the population composition).

We distinguished nine sociodemographic groups, as defined by the age classes introduced earlier and the differentiation by educational attainment of the young and mature working age population. We expect that the population aged 15−24 and the young working age population move into dense urban zones for educational and career development purposes. The mature working age population, by contrast, may look for places to raise children in socially desirable and affordable environments, situated in the less congested urban periphery. These sparse and ring areas may also be attractive to retirees looking for natural amenities, lower housing costs and well‐developed urban (transport) infrastructure. Figure 6 only includes the behavioural contributions to the crude net migration changes over time (e.g., the effect of changes in group‐specific rates, weighted by the groups' relative representation in the population), because these made up at least three quarters of the total contributions (contributions that represent less than 3% of the total variation are not shown to increase legibility of the figure).

The intensity of periurbanisation increased between the late 1960s and 1970s, as indicated by the generalised decline in net migration of dense and semidense areas. The declines were most marked (in precedence) among individuals aged 15−24, the children, the young working age population holding a postobligatory school diploma (i.e., secondary or tertiary level) and the mature working age population with a secondary education level. These groups increasingly favoured sparse and ring areas, thereby inflating net migration there. Between the late 1970s and 1980s, the behavioural changes were similar, except a reduced net migration among the working age population holding a postcompulsory education in the agglomeration rings.

During the shift towards re‐urbanisation between the late 1980s and 1990s, net migration in dense urban zones increased among individuals aged 15−24, the young working age population holding a tertiary education level and, to a lesser extent, those with a secondary level. Correspondingly, the migration balance of these groups declined in the remaining agglomeration density zones.

In the most recent period of resumed periurbanisation (2010−2018), the situation switched again. When compared to the late 1990s, net migration among children and the mature working age population holding a postcompulsory school diploma decreased in dense and semidense areas. The decline in the dense areas' crude rate was intensified by lower net migration among individuals aged 15−24 and, to a lesser extent, the young and low qualified working age population. These negative contributions were only partly compensated for by an increasing migratory balance among the young and highly skilled working age population, as well as among pensioners.

Correspondingly, the children, individuals aged 15−24, the young and low skilled working age population, as well as the mature working age population with postcompulsory education, moved to a larger extent into sparse and ring areas, when compared to the late 1990s, thereby inflating net migration levels there. The positive contributions to the crude migration balance in sparse zones and agglomeration rings have been only marginally counterbalanced by a lower inflow of pensioners and the mature working age population with a secondary education level. In short, periurbanisation has recently superseded re‐urbanisation mainly because the highly migratory youth and young adults head less frequently into dense areas, as well as because the out‐migration of families to the urban periphery intensified again (the rising net migration of the individuals aged 15−24 in semidense zones did not compensate for the other groups' negative impacts on the corresponding crude rate).

Differences across the urban hierarchy in terms of the main groups responsible for the changes in crude net migration of urban density zones only exist with regard to the dense areas (not shown). In the five largest agglomerations (Zurich, Basel, Geneva, Lausanne and Bern, with at least 300,000 inhabitants each in 2015), re‐urbanisation concerned the individuals aged 15−24 and the young and highly skilled working age population. In intermediate‐sized cities (with populations between 100,000 and 300,000), however, the process was initially driven only by the 15−24 old age group and, since 2010, also by pensioners. In small cities, re‐urbanisation resulted from higher net migration among the 15−24 old as well as among the young and mature working age population without tertiary education. However, the process was particularly short‐lived there, when compared to larger cities. It has completely disappeared in the last observation period. In all those city‐size classes, the renewed intensification of periurbanisation clearly outweighted the latest trend in re‐urbanisation.

## 5 SUMMARY AND DISCUSSION

Large‐scale periurbanisation since the second half of the 20th century not only affected population health, notably by transforming the urban mortality gradient (Lerch et al., 2017), but also environmental health and the conservation of biodiversity (McDonald et al., 2020). Yet recent developments in the underlying trends of internal migration have not attracted much attention by the research community. We have provided an exceptionally long‐term perspective of trends and spatial patterns of internal migration across the rural‐urban continuum of 79 urban agglomerations in Switzerland. This enabled us to assess whether the intensity of movements declined—as observed in an increasing number of countries around the world—and whether the recent re‐urbanisation—as noticed in several European countries—has consolidated. This would imply a slowing down and, ultimately, the end of periurbanisation. Using a very detailed disaggregation of the urban territory, we have ensured international comparability of the urban density zones' classification, as well as their consistency over time within the Swiss context.

Results reveal that the intensity of internal migration declined in the population aged 15−24 and, to a lesser extent, in the children and the elderly. This confirms previous observations at coarser spatial scales of the Swiss territory (Carnazzi‐Weber & Golay, 2005; Kupiszewski et al., 2000; Zufferey, 2020). Lower migration among the 15−24 old can be related to the decentralisation of the educational infrastructure, which limits the need for student migration from small cities or remote areas. The development of transport and communication technologies also increased the extent of commuting for work and study [from 12% to 20% of the population between 1990 and 2016; (BFS, 2018b)], as well as of home office work. This has to a certain extent substituted for a change in place of residence.

Declining migration among the elderly, by contrast, may be related to in‐situ urbanisation of their places of residence located in the urban periphery. Urban sprawl incorporated villages and smaller cities into expanding agglomerations. This improved the local availability of amenities and the transport connections to more central areas that are better deserved by public services. The decline in family migration, by contrast, mirrors a new sequencing of migratory and family events in people's life course. Rather than moving after the birth of their children, couples seem to move out of dense agglomerations zones into sparse areas in anticipation of their family formation plans—as observed in Finland (Kulu & Steele, 2013).

While migration in the life stage dedicated to education is declining, spatial mobility increasingly matters in the subsequent professional life. The population in working ages moves more and more over time—whatever the educational attainment. With the additional diffusion of tertiary education in society, the highly skilled (and generally more migratory) subgroup is increasingly represented in the working age population, thereby inflating its crude rate of internal migration—as observed in Australia (Kalemba et al., 2021). In Switzerland, this rising trend in migration can be related, on the one hand, to the postponement of crucial life course transitions, which deferred life‐adjusting moves to higher adult ages. Young people spend more time in education before entering the labour market, and the average age of women at their first birth increased from 25 to 31 years between 1970 and 2016 (BFS, 2018a). On the other hand, increased spatial mobility in combination with professional mobility is likely to increase wages, the prospects for career progression and reflects the increasing requirement of postindustrial labour markets in terms of workers' employment flexibility.

The Swiss‐wide pattern of internal migration by agglomeration density zone confirms that the country reached an advanced stage in the process of population redistribution. While the rural exodus and the dominant intercity movements declined, migration is more and more confined within a given agglomeration. Results also reveal a clear shift towards increasingly centrifugal flows within and across agglomerations over the last 50 years. We traced the onset of this periurbanisation process back to the late 1960s in the two largest agglomerations of Switzerland. The phenomenon subsequently diffused to virtually all other cities until 1990.

Results also confirmed the emergence of re‐urbanisation at the turn of the 21st century. However, this shifting geography of population movements towards a centripetal pattern concerned only some agglomerations and specific sociodemographic groups. At the same time, the process of periurbanisation continued to unfold and remained the dominant form of population redistribution in most Swiss cities. Re‐urbanisation can be related, on the one hand, to the educational expansion at the turn of the new Century. This has pulled the youth into city centres, where the higher‐level educational infrastructure is concentrated. On the other hand, city centres are also attractive for the young and highly skilled adults in working age, who are developing their professional career and would like to benefit from a vibrant city life, while being still childless. More generally, the late 1990s and early 2000s constitute a particular period marked by major urban renewal projects in dense agglomeration zones that led to the substitution of modern apartment complexes to industrial brownfields. Thus, residential space for new inhabitants temporarily increased.

Yet re‐urbanisation was a passing phase. Periurbanisation recently intensified again and increasingly focuses on the rural rings around agglomerations—rather than on the sparse urban zones closer to the centres. This may be explained by the soaring rental prices in Switzerland (which increased by at least 31% since 2000 according to the SFSO's rental price index), as observed in Germany (Osterhage, 2018; Sander, 2014; Stawarz et al., 2021). In Switzerland, gentrification particularly concerned the dense zones in large agglomerations (Rérat et al., 2010). Our results suggest that the 15−24 old, the young and low‐skilled working age population, as well as families, have been ‘evicted’ from dense zones to other more affordable residential areas in the urban periphery. This interpretation is plausible with regard to similar observations in other European countries (Dembski et al., 2021).

At the same time, the highly skilled working age population continues to afford a relocation to dense urban zones. Thus, agglomeration centres more and more constitute dynamic places of entertainment and elevator regions for young workers on an ascending professional track (Fielding, 1993): being in the hubs of the Swiss economy helps them better capitalising their skills to acquire the resources necessary to find a high quality residential environment in later (family) stages of their life course. In addition, the elderly emerge as a new subgroup valuing dense residential zones in intermediate‐sized cities probably because amenities are less developed in these urban peripheries, when compared to the fringes of larger agglomerations.

In conclusion, periurbanisation in Switzerland did not come to a halt. The sprawl of the urban population and the built‐up environment not only expanded spatially into more distant rural areas over time. Centripetal movements also increased in intensity among the working age population. Although re‐urbanisation indeed emerged among the highly educated and young working age population and (more recently) the pensioners, period effects such as urban revitalisation programmes and the diffusion of higher‐level education temporarily inflated its demographic importance in Switzerland. Subsequent gentrification of dense urban areas reverted the trend in population redistribution back to urban deconcentration. This responsiveness of internal migration to periodic circumstances reminds us of the complexity of the phenomenon, as driven by multiple and often unforeseeable factors.

A methodological note on the discrepancy between Rérat's (2012, 2019) positive assessment of the Swiss trend in re‐urbanisation and our contradicting results is warranted. Our time series are not confounded by boundary changes and reclassification of municipalities over time (as we harmonised the administrative settlement structure). We rely on another (internationally comparable) classification of agglomeration areas, while accounting for an additional agglomeration zone (e.g., the rural ring). Rather than analysing trends in total population growth, as Rérat did, we have focused on the spatial redistribution of Switzerland's resident population to better understand how denser and sparser agglomeration zones are affected in a relational perspective. We have also neglected international migration flows (but not the internal movements of the immigrant stock). The historical peak levels and the highly‐skilled profile of international inflows since the turn of the new century can be interpreted as yet another period effect that temporarily inflated the renewed population growth in dense zones. More research is needed to better understand the role of international immigration and emigration alongside their interactions with internal movements in the process of city growth. Potential scenarios are multiple. A continuously large international inflow may be necessary to compensate for the immigrant stock's international re‐emigration from Switzerland and its periurbanisation within the country.

Our results have implications for Switzerland's urban structure. With renewed intensification of periurbanisation, the country is likely to become one single mega‐urban region soon—rather than a set of interacting, densely populated but spatially distinct metropolitan areas. Agglomeration ring areas will constitute urbanised interstices between formerly separated agglomerations and will become ever more attractive locations for dual‐earner couples working in adjacent cities. Despite recent official directives that aimed at limiting urban sprawl, many municipalities located in agglomeration ring areas have already consumed all the allotted constructible land for the next 20 years. If local authorities really want to reverse the current trend and limit negative externalities of urban sprawl on the environment and population health, they need to intervene more strongly in the zoning and housing market. Given the decentralised institutional structure of Switzerland, more cooperation between administrative units would help imposing urban containment measures, which are challenged by competition for tax payers. Moreover, to ensure inclusive and sustainable densification of cities, social mixing of the habitat is key. The construction of social housing and housing cooperatives in central areas of agglomerations can avoid the eviction of individuals who aspire to live in the city and limit urban sprawl.

## Data Availability

The data that support the findings of this study are available from Swiss Federal Statistical Office. Restrictions apply to the availability of these data, which were used under license for this study. Data are available from the author(s) with the permission of Swiss Federal Statistical Offfice.

## Footnotes

1 Heat island effects refer to the stronger warming up of air masses on impervious surfaces (when compared to natural surfaces) due to limited reradiation of heat and evapotranspiration of trees, as well as increased traffic and energy consumption.

2
https://www.eda.admin.ch/sdg11-e

3 Urban sprawl manifests itself in different forms—such as through population distribution, the geography of population densities or land use patterns. In this paper, we focus on the underlying demographic behaviours, as constituted by internal migrations of the resident population.

4 This so‐called re‐urbanisation process does not exclusively refer to population growth after a period of decline in central zones of agglomeration. Alternative perspectives in the literature include a final stage in the spatial cycle of urban growth (in which central areas grow more than the urban agglomeration belts), or the qualitative diversification of urban society [see Rérat (2012) for a theoretical discussion]. As stated above, we focus on the intensity and changing geography of internal migration that underly these multidimensional transformations.

5 There is virtually no information on the quality of information on usual place of residence in censuses. Uncertainty may be high, especially for data on the youth. In the municipality of Zurich, for example, the net migration rate of the population aged 20−24 years increased sharply from 2.82% in 1986−1990 to 21.12% in 1996−2000. However, rather than decreasing in 2010−2018 (because weekly residence is not considered in the register data), the rate further increased to 29.14% in 2010−2018, even though the percentage of weekly residents dropped from 28% to 24% between 2000 and 2018 (according to data from Statistical Office of the municipality of Zurich). At the censuses 1990 and 2000, many young people may have indicated as their economic residence the place in which they were registered and have grown up, even though they spend parts of the week elsewhere.

## References

[psp2621-bib-0001] Alvarez, M. , Bernard, A. , & Lieske, S. N. (2021). Understanding internal migration trends in OECD countries. Population, Space and Place, 27(7), e2451. 10.1002/psp.2451

[psp2621-bib-0002] Bell, M. , Charles‐Edwards, E. , Bernard, A. , & Ueffing, P. (2018). Global trends in internal migration. In T. Champion , T. Cooke , & I. Shuttleworth (Eds.), Internal migration in the developed world. Routledge.

[psp2621-bib-0003] Bell, M. , Charles‐Edwards, E. , Ueffing, P. , Stillwell, J. , Kupiszewski, M. , & Kupiszewska, D. (2015). Internal migration and development: Comparing migration intensities around the world. Population and Development Review, 41(1), 33–58. 10.1111/j.1728-4457.2015.00025.x

[psp2621-bib-0004] Bernard, A. , & Bell, M. (2018). Educational selectivity of internal migrants: A global assessment. Demographic research, 39(29), 835–854. 10.4054/DemRes.2018.39.29

[psp2621-bib-0005] BFS . (2017). *Gemeindetypologie und Stadt/Land‐Typologie 2012*. Bundesamt für Statistik.

[psp2621-bib-0006] BFS . (2018a). Geburtenhäufigkeit: Situation 2016 und Tendenzen. BFS Aktuel, 2018, 4.

[psp2621-bib-0007] BFS . (2018b). Pendlermobilität in der Schweiz 2016. BFS Aktuel, 2018, 12.

[psp2621-bib-0008] BFS . (2019). *Die Bodennutzung in der Schweiz—Resultate der Arealstatistik 2018*. Bundesamt für Statistik (BFS).

[psp2621-bib-0009] BFS . (2021). *Historisiertes Gemeindeverzeichnis der Schweiz*. Bundesamt für Statistik.

[psp2621-bib-0010] Carnazzi‐Weber, S. , & Golay, S. (2005). *Interne migration in der Schweiz*. Office fédéral de la statistique.

[psp2621-bib-0011] Champion, A. G. (1989). Counterurbanization: The changing pace and nature of population deconcentration. Edward Arnold.

[psp2621-bib-0012] Champion, A. G. (2001). Urbanization, suburbanization, counterurbanization and reurbanization. In R. Paddison (Ed.), Handbook of urban studies (pp. 143–161). SAGE Publications.

[psp2621-bib-0013] Charles‐Edwards, E. , Bell, M. , Bernard, A. , & Zhu, Y. (2017). Internal migration in the countries of Asia: Levels, ages, and spatial impacts. ADRI Working Paper, 2017(001), 41. 10.1080/17441730.2019.1619256

[psp2621-bib-0014] Charton, L. , & Wanner, P. (2001). *Migrations internes et changements familiaux en Suisse: Analyse du module “mobilité” de l'enquête suisse sur la population active de 1998*. Office fédéral de la statistique.

[psp2621-bib-0015] Cooke, T. J. (2013). Internal migration in decline. The Professional Geographer, 65(4), 664–675. 10.1080/00330124.2012.724343

[psp2621-bib-0016] Cooke, T. J. , Wright, R. , & Ellis, M. (2018). A prospective on Zelinsky's hypothesis of the mobility transition. Geographical Review, 108(4), 503–522. 10.1111/gere.12310 32494088 PMC7269166

[psp2621-bib-0017] Cunha, A. D. , & Both, J.‐F. (2004). *Métropolisation, villes et agglomérations—Structure et dynamiques socio‐démographiques des espaces urbains* (Recensement Fédéral de La Population 2000, p. 103). Office fédéral de la statistique.

[psp2621-bib-0018] Dembski, S. , Sykes, O. , Couch, C. , Desjardins, X. , Evers, D. , Osterhage, F. , Siedentop, S. , & Zimmermann, K. (2021). Reurbanisation and suburbia in northwest Europe: A comparative perspective on spatial trends and policy approaches. Progress in Planning, 150, 100462. 10.1016/j.progress.2019.100462

[psp2621-bib-0019] Fielding, T. (1993). Migration and the metropolis: An empirical and theoretical analysis of inter‐regional migration to and from South East England. Progress in Planning, 39, 75–166. 10.1016/0305-9006(93)90006-F

[psp2621-bib-0020] Fioretta, J. , & Wanner, P. (2017). Rester ou partir? Les déterminants des flux d'émigration récents depuis la Suisse. Revue européenne des migrations internationales, 33(1), 111–131. 10.4000/remi.8532

[psp2621-bib-0021] Geyer, H. S. , & Kontuly, T. (1993). A theoretical foundation for the concept of differential urbanization. International Regional Science Review, 15(2), 157–177. 10.1177/016001769301500202

[psp2621-bib-0022] de Haas, H. (2010). Migration and development: A theoretical perspective. International Migration Review, 44(1), 227–264. 26900199 10.1111/j.1747-7379.2009.00804.x PMC4744987

[psp2621-bib-0023] Halbac‐Cotoara‐Zamfir, R. , Egidi, G. , Mosconi, E. M. , Poponi, S. , Alhuseen, A. , & Salvati, L. (2020). Uncovering demographic trends and recent urban expansion in metropolitan regions: A paradigmatic case study. Sustainability, 12(9), 3937. 10.3390/su12093937

[psp2621-bib-0024] Kabisch, N. , & Haase, D. (2011). Diversifying European agglomerations: Evidence of urban population trends for the 21st century: Diversifying european agglomerations. Population, Space and Place, 17, 236–253. 10.1002/psp.600

[psp2621-bib-0025] Kalemba, S. V. , Bernard, A. , Charles‐Edwards, E. , & Corcoran, J. (2021). Decline in internal migration levels in Australia: Compositional or behavioural effect. Population, Space and Place, 27(7), e2341. 10.1002/psp.2341

[psp2621-bib-0026] Kaufmann, V. (2012). La sociologie urbaine en Suisse: Histoire, développement, débats actuels. SociologieS [Enligne]. consulté le octobre 12, 2022. https://journals.openedition.org/sociologies/4189

[psp2621-bib-0027] Kitagawa, E. M. (1955). Components of a difference between two rates. Journal of the American Statistical Association, 50(272), 1168–1194. 10.2307/2281213

[psp2621-bib-0028] Kontuly, T. , & Geyer, H. S. (2003). Lessons learned from testing the differential urbanisation model. Tijdschrift Voor Economische en Sociale Geografie, 94(1), 124–128. 10.1111/1467-9663.00242

[psp2621-bib-0029] Kulu, H. , & Steele, F. (2013). Interrelationships between childbearing and housing transitions in the family life course. Demography, 50(5), 1687–1714. 10.1007/s13524-013-0216-2 23703223

[psp2621-bib-0030] Kupiszewski, M. , Schuler, M. , Reichle, M. , Durham, H. , & Rees, P. (2000). Internal migration and regional population dynamics in, Europe: Switzerland case study (working paper). School of Geography, University of Leeds. https://eprints.whiterose.ac.uk/5026/

[psp2621-bib-0031] Lerch, M. , & Wanner, P. (2010). Quels apports démographiques des migrations internes des étrangers pour les villes suisses? GeoRegard, 2010(3), 91–147. 10.33055/GEOREGARDS.2010.003.01.73

[psp2621-bib-0032] Lerch, M. , Wanner, P. , & Oris, M. (2017). Periurbanization and the transformation of the urban mortality gradient in Switzerland. Population‐E, 72(1), 93–122.

[psp2621-bib-0033] McDonald, R. I. , Mansur, A. V. , Ascensão, F. , Colbert, M. , Crossman, K. , Elmqvist, T. , Gonzalez, A. , Güneralp, B. , Haase, D. , Hamann, M. , Hillel, O. , Huang, K. , Kahnt, B. , Maddox, D. , Pacheco, A. , Pereira, H. M. , Seto, K. C. , Simkin, R. , Walsh, B. , … Ziter, C. (2020). Research gaps in knowledge of the impact of urban growth on biodiversity. Nature Sustainability, 3(1), 16–24. 10.1038/s41893-019-0436-6

[psp2621-bib-0034] OECD . (2018). Rethinking urban sprawl: Moving towards sustainable cities. OECD Publishing.

[psp2621-bib-0035] Osterhage, F. (2018). The end of reurbanisation? Phases of concentration and deconcentration in migratory movements in North Rhine‐Westphalia. Comparative Population Studies, 43. 10.12765/CPoS-2018-10

[psp2621-bib-0036] Rees, P. , Bell, M. , Kupiszewski, M. , Kupiszewska, D. , Ueffing, P. , Bernard, A. , Charles‐Edwards, E. , & Stillwell, J. (2017). The impact of internal migration on population redistribution: An international comparison. Population, Space and Place, 23(6), e2036. 10.1002/psp.2036

[psp2621-bib-0037] Rérat, P. (2012). The new demographic growth of cities—The case of reurbanisation in Switzerland. Urban Studies, 49(5), 1107–1125. 10.1177/0042098011408935

[psp2621-bib-0038] Rérat, P. (2019). The return of cities: The trajectory of Swiss cities from demographic loss to reurbanization. European Planning Studies, 27(2), 355–376. 10.1080/09654313.2018.1546832

[psp2621-bib-0039] Rérat, P. , Söderström, O. , Piguet, E. , & Besson, R. (2010). From urban wastelands to new‐build gentrification: The case of Swiss cities. Population, Space and Place, 16(5), 429–442. 10.1002/psp.595

[psp2621-bib-0040] Rodriguez, J. (2007). *Spatial distribution of the population, internal migration and development in Latin America and the Caribbean*. *United Nations Expert Group Meeting on Population Distribution, Urbanization, Internal Migration and Development, New York, 21−23 January 2008, UN/POP/EGM‐URB/2008/06*, 30.

[psp2621-bib-0041] Rubiera‐Morollón, F. , & Garrido‐Yserte, R. (2020). Recent literature about urban sprawl: A renewed relevance of the phenomenon from the perspective of environmental sustainability. Sustainability, 12(16), 6551. 10.3390/su12166551

[psp2621-bib-0042] Salvati, L. , Serra, P. , Bencardino, M. , & Carlucci, M. (2019). Re‐urbanizing the European city: A multivariate analysis of population dynamics during expansion and recession times. European Journal of Population, 35(1), 1–28. 10.1007/s10680-017-9462-0 30976266 PMC6357255

[psp2621-bib-0043] Sander, N. (2014). Internal migration in Germany, 1995−2010: New insights into East‐West migration and re‐urbanisation. Comparative Population Studies, 39(2), 217–246. 10.12765/CPoS-2014-04

[psp2621-bib-0044] Shaw, B. J. , van Vliet, J. , & Verburg, P. H. (2020). The peri‐urbanization of Europe: A systematic review of a multifaceted process. Landscape and Urban Planning, 196, 103733. 10.1016/j.landurbplan.2019.103733

[psp2621-bib-0045] Sjaastad, L. A. (1962). The costs and returns of human migration. Journal of Political Economy, 70(5), 80–93.

[psp2621-bib-0046] Skeldon, R. (2019). A classic re‐examined: Zelinsky's hypothesis of the mobility transition. Migration Studies, 7(3), 394–403. 10.1093/migration/mny019

[psp2621-bib-0047] Stawarz, N. , Sander, N. , & Sulak, H. (2021). Internal migration and housing costs—A panel analysis for Germany. Population, Space and Place, 27(4), e2412. 10.1002/psp.2412 PMC9110994 35601664

[psp2621-bib-0048] Wanner, P. (2020). Migration internationale et intégration dans une perspective longitudinale, *Panorama de la société suisse 2020: Migration–Intégration–Participation* (p. 112). Office Fédéral de la Statistique.

[psp2621-bib-0049] Wanner, P. , & Steiner, I. (2018). Une augmentation spectaculaire de la migration hautement qualifiée en Suisse. Social Change in Switzerland No. 16. Retrieved from https://wwww.socialchangeswitzerland.ch

[psp2621-bib-0050] Zelinsky, W. (1971). The hypothesis of the mobility transition. Geographical Review, 61(2), 219–249. 10.1111/gere.12310 PMC7269166 32494088

[psp2621-bib-0051] Zufferey, J. (2020). Les migrations internes en Suisse: Pratiques et impacts, Migration—Intégration—Participation (pp. 86–95). Office fédéral de la statistique.

